# Children's identification of familiar songs from pitch and timing cues

**DOI:** 10.3389/fpsyg.2014.00863

**Published:** 2014-08-06

**Authors:** Anna Volkova, Sandra E. Trehub, E. Glenn Schellenberg, Blake C. Papsin, Karen A. Gordon

**Affiliations:** ^1^Department of Psychology, University of Toronto MississaugaMississauga, ON, Canada; ^2^Department of Otolaryngology, University of TorontoToronto, ON, Canada

**Keywords:** song identification, children, cochlear implants, pitch, rhythm, melody

## Abstract

The goal of the present study was to ascertain whether children with normal hearing and prelingually deaf children with cochlear implants could use pitch or timing cues alone or in combination to identify familiar songs. Children 4–7 years of age were required to identify the theme songs of familiar TV shows in a simple task with excerpts that preserved (1) the relative pitch and timing cues of the melody but not the original instrumentation, (2) the timing cues only (rhythm, meter, and tempo), and (3) the relative pitch cues only (pitch contour and intervals). Children with normal hearing performed at high levels and comparably across the three conditions. The performance of child implant users was well above chance levels when both pitch and timing cues were available, marginally above chance with timing cues only, and at chance with pitch cues only. This is the first demonstration that children can identify familiar songs from monotonic versions—timing cues but no pitch cues—and from isochronous versions—pitch cues but no timing cues. The study also indicates that, in the context of a very simple task, young implant users readily identify songs from melodic versions that preserve pitch and timing cues.

## Introduction

Melodies are defined by pitch relations in a linear succession of musical tones (*contour* and *intervals*) and their temporal organization (*meter* and *rhythm*). For Western adults with normal hearing (NH), pitch patterns are better cues to the identification of familiar songs than are temporal patterns (Hébert and Peretz, 1997), perhaps because pitch patterns in Western music are more complex and distinctive than temporal patterns (Prince et al., 2009). Little is known about the separate contributions of pitch and timing information to children's identification of familiar songs because these parameters have not been manipulated independently. What is clear is that children are sensitive to a variety of musical features including scale, contour, key, rhythm, and tempo (Trehub et al., 1985; Pick et al., 1988; Trehub and Thorpe, 1989; Drake et al., 1990).

Because young children engage in more holistic or integrated processing of pitch and timing cues compared to older children and adults (e.g., Overy et al., 2004), they may have difficulty identifying familiar songs from pitch or temporal patterns alone. When 5- to 8-year-old children with normal hearing are presented with familiar songs that retain the pitch and temporal properties of the canonical versions but lack the vocals and instrumentation, they identify the songs much less accurately than adults do (Vongpaisal et al., 2006). As for song production, children reportedly master the temporal structure before mastering the contour and intervals, using the lyrics and speech rhythms as anchoring cues (Welch et al., 1998; Rutkowski and Miller, 2003).

Deaf individuals who use cochlear implants (CIs) present an interesting case with respect to music perception and memory because their prostheses, which are designed to optimize speech reception, provide reasonable temporal information but degraded spectral information (Geurts and Wouters, 2001; Smith et al., 2002; Gates and Miyamoto, 2003). Relatively accurate speech perception is possible under conditions of severe spectral degradation (Shannon et al., 1997), but such degradation has particularly adverse consequences for music perception (Cooper et al., 2008; Kang et al., 2009). Presumably, music heard through such devices would sound unpleasant, as reported by many CI users who became deaf as adolescents or adults (Gfeller et al., 2000; Lassaletta et al., 2007). By contrast, prelingually deaf children with CIs, who have never heard music acoustically, claim to like music and often participate in musical activities (Nakata et al., 2006; Vongpaisal et al., 2006). In any case, one would expect them to be like adult CI users in according greater priority to temporal than to pitch patterns in music and speech (Fu, 2002; Gfeller et al., 2005).

The pitch patterning of speech provides information about a speaker's emotions and distinguishes statements from yes/no questions. Although such pitch variations are large relative to those encountered in music (Fitzsimmons et al., 2001), adult and child CI users are much poorer than their NH peers at identifying the intentions expressed through intonation, or speech melody (Chatterjee and Peng, 2007; Peng et al., 2008; Nakata et al., 2012; Volkova et al., 2013). Not surprisingly, the small pitch steps and precise pitch relations of music (Vos and Troost, 1989) pose even greater challenges for CI users (McDermott, 2004; Drennan and Rubinstein, 2008), who may detect small differences between single pitches but are generally unable to differentiate brief melodies when one tone is shifted by one or two semitones (Vongpaisal et al., 2006; Galvin et al., 2007; Cooper et al., 2008; Hopyan et al., 2012).

Identifying the direction of pitch movement is also a commonly reported difficulty, especially when the pitch steps are small. For example, adult CI users' ability to rank the second of two pitches as higher or lower than the first typically requires differences of three or more semitones (Gfeller et al., 2007; Kang et al., 2009; Sucher and McDermott, 2009). It is possible that sensations arising from melodies may be markedly different for CI users, perhaps with pitch changes heard as changes in timbre (McDermott, 2004; Moore and Carlyon, 2005).

As noted, temporal patterns in speech and music are more accessible than pitch patterns for implant users. Child CI users differentiate same-gender talkers on the basis of subtle timing differences in articulation and global differences in speech rhythm and speaking rate (Vongpaisal et al., 2010). Adult CI users' ability to perceive musical tempo and rhythm is similar to that of NH listeners (Gfeller and Lansing, 1991; Gfeller et al., 1997; Kong et al., 2004; Cooper et al., 2008). Nevertheless, adult and child CI users' recognition of melodies is considerably poorer than that of NH listeners even in the presence of distinctive timing cues (Gfeller et al., 2002, 2005; Stordahl, 2002; Vongpaisal et al., 2006, 2009; Nimmons et al., 2007). Moreover, child CI users' song production skills reveal age-appropriate temporal patterning but severely deficient pitch patterning (Nakata et al., 2006; Xu et al., 2009). In short, the available evidence confirms that timing cues make a greater contribution to speech and music processing for CI users than they do for NH listeners.

Although timing cues facilitate melody perception in CI users, it is unclear whether they would be sufficient for the identification of familiar melodies, both for children with normal hearing and for CI users. Little is known about CI users' long-term representations of familiar music because comparisons of melody recognition with and without timing cues typically preserve the original pitch patterns (Kong et al., 2004; Galvin et al., 2007; Nimmons et al., 2007; Hsiao, 2008). In fact, no study to date has compelled children, hearing or deaf, to rely entirely on timing cues by using stimuli with unchanging pitch and timbre.

Some pitch patterns seem to be accessible to CI users in speech and musical contexts. For example, child CI users achieve modest success in differentiating Cantonese lexical tones (Barry et al., 2002), perhaps by capitalizing on temporal envelope cues (Fu et al., 1998). Moreover, extended training (1 week to 2 months) generates improvement in adult CI users' identification of musical contours (e.g., flat, rising, falling) and familiar melodies (Galvin et al., 2007).

In the present investigation, we asked whether young CI users and NH listeners could use pitch or timing cues separately or in combination to identify familiar melodies. In general, studies of familiar melody identification with CI users choose tunes that are well known to the general population but not necessarily to the CI users under consideration. Such stimulus selection may contribute to adult CI users' inability to recognize many “familiar” recordings (Gfeller et al., 2005). Prelingually deaf CI users 8–18 years of age report familiarity with 50% of well known children's or folk songs, yet they identify less than half of the “familiar” songs (closed-set task) from excerpts that preserve the original pitch and temporal properties (Stordahl, 2002; Olszewski et al., 2005). With familiar songs that have canonical renditions such as pop recordings, child and adolescent CI users can identify the songs from instrumental versions that preserve the original instrumentation, pitch level, and timing (i.e., without the lyrics) but not from simple piano renditions of the main melody (Vongpaisal et al., 2006).

The television programs that children watch regularly provide a rich source of familiar musical materials. In fact, child CI users can identify the theme songs of their favorite television programs (Mitani et al., 2007; Vongpaisal et al., 2009). In one study, CI users 4–8 years of age (mean age of 6.5 years) identified the songs when the original cues (instrumental and vocal) were intact but not otherwise (Mitani et al., 2007). In another study, CI users 5–11 years of age (mean age of 8.4 years) showed above-chance identification of multiple-instrument versions (original minus words) and of monophonic flute versions of TV theme songs with intact pitch and timing cues, but they performed much more poorly (approximately 37% correct, chance level of 25%) than on the original versions (65% correct) (Vongpaisal et al., 2009). In all cases, the performance of child CI users was well below that of their NH peers. Differences in performance levels between the two studies with TV theme music could stem from age-related cognitive differences, implant experience, and differential exposure to the music.

We sought to minimize the cognitive demands on participants in the present study, who were comparable in age to those in Mitani et al. (2007). We opted for two response alternatives with feedback, rather than the three or more alternatives in previous studies of familiar song recognition with child CI users, because melody recognition in earlier studies was barely above chance (Olszewski et al., 2005; Vongpaisal et al., 2009) or at chance levels (Vongpaisal et al., 2006; Mitani et al., 2007) when pitch and timing cues were intact. Several studies of emotion identification in speech and music by preschool or school-age children have used two response alternatives such as happy and sad (Dalla Bella et al., 2001; Mote, 2011; Volkova et al., 2013), sometimes with reinforcement or feedback (e.g., Morton et al., 2003; Volkova et al., 2013) to provide encouragement or guidance to young children. Our participants consisted of child CI users 5–7 years of age and NH children who were comparable in years of functional hearing and socioeconomic status. The children were required to identify theme songs from familiar television programs in the presence or absence of various cues.

Three conditions were of principal interest. In one, the melody, which was sung in the original TV versions, was presented in a synthesized flute timbre with pitch and temporal patterns preserved, as in the melody condition of Mitani et al. (2007) and Vongpaisal et al. (2009). Essentially, this condition provided a baseline for the evaluation of performance in the two more challenging conditions. In a second condition, which had not been evaluated previously, the original tempo and rhythm were preserved but pitch cues were removed by using a percussion instrument (woodblock) with unchanging and non-salient pitch. In a third condition that has been evaluated in adults (e.g., Hébert and Peretz, 1997; Kang et al., 2009) and in a single study with Mandarin-speaking children (Hsiao, 2008), the relative pitch patterns (i.e., melodic contour and intervals) were preserved and presented in a synthesized flute timbre, but timing cues were removed by having all notes (and inter-onset intervals) of equal duration. Isochronous versions of familiar melodies are often created by replacing sustained, or long-duration notes, with repeated short-duration notes (Nimmons et al., 2007; Kang et al., 2009), which eliminates grouping cues but preserves tempo and meter. By contrast, the isochronous melodies in the present experiment were created by replacing long-duration notes with single short-duration notes, so that the number of notes remained unchanged but the tempo, meter, and overall duration were altered. This reduction of cues was expected to increase the difficulty of song recognition.

The conditions of greatest interest were those with timing-only or pitch-only cues, which would indicate whether either cue on its own enables children to identify familiar melodies. In the melody, timing-only, and pitch-only conditions, all notes were of equal amplitude (same MIDI velocity). We first confirmed, however, that the children could identify the theme songs in their original vocal/instrumental form (i.e., as presented on television) or in similar form but without the lyrics (following Mitani et al., 2007; Vongpaisal et al., 2009). The conditions were presented in fixed order, corresponding to the expected order of increasing difficulty for CI users: original versions first, followed by instrumental, melodic, timing-only, and pitch-only versions. Fixed order, from least to greatest difficulty, has been used in a number of studies with child CI users (e.g., Vongpaisal et al., 2006; Mitani et al., 2007). Obviously, prior presentation of the original versions would prime children's recognition of the versions with reduced cues. In this instance, good performance on the melody versions, which has been difficult to obtain with child CI users (Mitani et al., 2007; Vongpaisal et al., 2009), was essential for evaluating performance on timing-only and pitch-only versions. In principle, children could perform worse on later conditions because of fatigue or boredom rather than task difficulty, but interactive computer tasks with familiar television cartoon characters (Mitani et al., 2007; Vongpaisal et al., 2009; van Heutgen et al., 2014) tend to be highly engaging for young children.

## Materials and methods

### Participants

The participants included eight bilateral CI users (4 girls and 4 boys, *M* = 6.2 years, *SD* = 0.7; range: 5.1–7.2) who were from middle-class families in a large metropolitan area (for background information, see Table 1). Bilateral implants are currently a popular option in this geographic region for children like the seven in our sample who were congenitally or prelingually deaf but with functional auditory nerves. One child (CI-4) had a progressive hearing loss from birth. All CI users had Nucleus 24 Contour and/or Nucleus Freedom Contour Advance implants programmed with the Advanced Combination Encoder (ACE) processing strategy, with at least 4 years of implant experience (*M* = 5.0 years; *SD* = 0.6; range: 4.0–5.9). Their age range was selected for comparability with that of Mitani et al. (2007). When tested with their CIs, the children in our sample could detect tones in the speech range within normal limits (10–30 dB HL).

**Table 1:** **CI Participant information**.

**Participant**	**Gender**	**Age at test (years)**	**Age at activation (years)**	**Etiology**
CI-1	M	5.7	0.8; 1.7	Genetic
CI-2	M	5.5	1.1; 1.1	Genetic
CI-3	F	6.8	1.0; 3.6	Genetic
CI-4^*^	F	7.2	2.5; 4.0	Unknown
CI-5	M	5.8	0.9; 1.8	Genetic
CI-6	M	6.3	0.8; 1.5	Genetic
CI-7	F	5.1	1.1; 1.1	Genetic
CI-8	F	6.9	1.0; 3.5	Unknown

* *Progressive hearing loss from birth*.

All of the CI users had participated in auditory-verbal therapy for 2 or more years after activation of their implants, they communicated exclusively by auditory-oral means (i.e., no sign language), and they were in age-appropriate school classes with their NH peers. At the time of testing, one CI user (CI-6) had been taking private piano lessons for approximately 2 years, and another (CI-1) for approximately 4 months. Two other CI users (CI-7 and CI-8) had no formal music training but they were participating in extracurricular choral activities at school. The remaining four CI users had no participation in extracurricular musical activities at school or in the community. A comparison sample of 16 NH children was matched roughly to the CI users on “hearing age” or years of auditory experience (*M* = 5.1 years, *SD* = 0.6, range: 4.3–6.3) and socioeconomic status. The hearing of NH children was not tested, but parents reported that their children had no personal or family history of hearing problems and all were free of colds on the day of testing. No NH child had formal music training.

This research received ethical approval from our institution and conformed to accepted ethical standards for the treatment of human participants.

## Apparatus and stimuli

Testing took place in a double-walled sound-attenuating booth at a university laboratory or comparable hospital laboratory, at the parents' convenience. At the university, a computer workstation and amplifier (Harman/Kardon HK3380) outside the booth were linked to a 17-in touch-screen monitor (Elo LCD Touch Systems) and two high-quality loudspeakers (Electro-Medical Instrument Co.) inside the booth. At the hospital facility, a GSI 61 two-channel clinical audiometer (Grason-Stadler Instruments) replaced the amplifier. The loudspeakers were on either side of the child, at 45° azimuth and an approximate distance of 80 cm, while the child faced the touch-screen monitor. Customized software presented stimuli and recorded responses when the child touched the screen. The experimenter used a portable keyboard for the few children who preferred to point to their on-screen choices without actually touching them. Stimuli were played at approximately 65 dB SPL.

The eight television shows chosen by children, along with their theme songs, are shown in Table 2. The 40 stimuli consisted of an excerpt from each theme song, with each excerpt presented in 5 different versions. The first two conditions (*original* and *instrumental*) served to confirm that children could readily identify the songs with all or most features intact. The original versions were taken directly from theme songs played at the beginning of popular children's TV programs by re-recording the audio tracks as digital sound files. The instrumental versions, which were created by a professional music technician, duplicated the original multi-instrument accompaniment but replaced the sung portions (i.e., the sung melody with lyrics) with a synthesized flute (following Mitani et al., 2007; Vongpaisal et al., 2009). The resulting versions matched the pitch, rhythm, tempo, and timbre of the original recordings except for the absence of the voice. The other three conditions (*melodic, timing-only*, and *pitch-only*) were of principal interest. The melodic versions consisted of the flute melodies from the instrumental condition (i.e., the single melody line sung in the original version), presented in the original tempo and key but without accompaniment. The synthesized flute melody in the instrumental and melodic versions of one song (from *Dora the Explorer*) was approximately one octave higher than the others, as it is in the original recording.

**Table 2:** **Key, pitch range, and original tempo of melodies from the TV theme songs**.

**Show/song**	**Key**	**Pitch range (Hz)**	**Tempo (bpm)**
Dora the Explorer	C major	C5-A5 (523–880) C4-A4 (262–440), pitch-only	107
Diego	E major	D#4-C#5 (311–554)	118
Backyardigans	D major	D4-D5 (293–587)	95
Franklin	D major	F#4-F#5 (369–738)	94
Hannah Montana	Db major	Bb3-Bb4 (233–466)	124
Suitelife on Deck	C major	C4-A4 (261–440)	108

**Figure 1:**
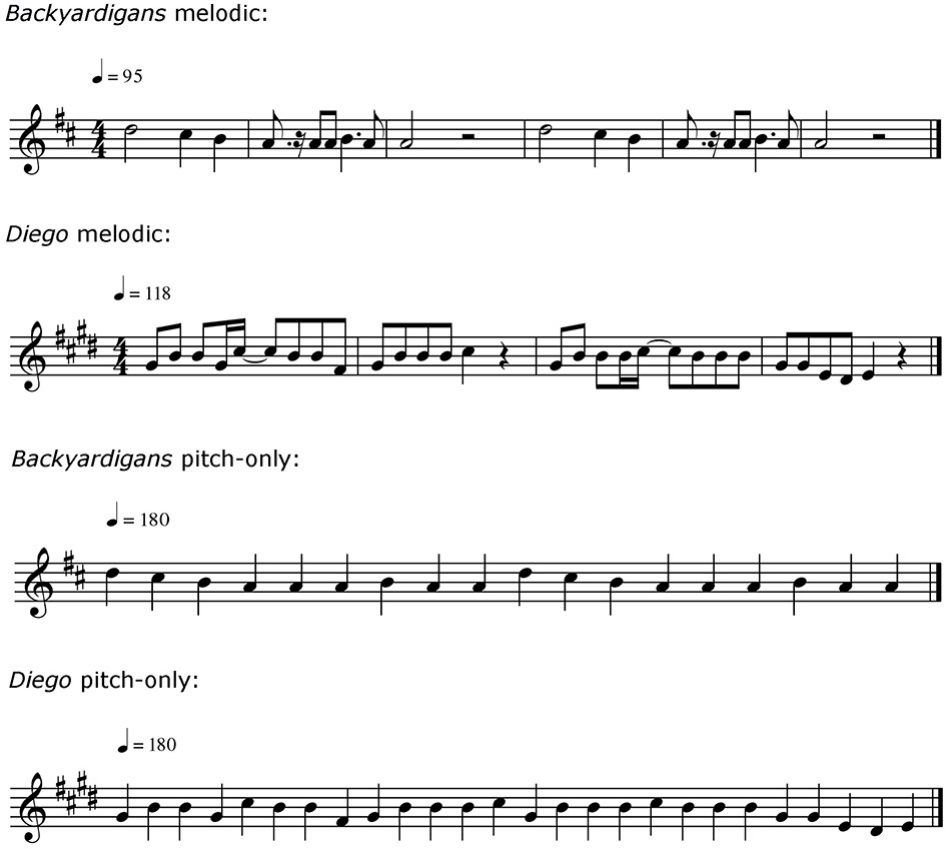
**Sample melodic and pitch-only versions of two of the test songs**.

Timing-only and pitch-only versions were created with Finale 2009 software (MakeMusic Inc., 2008) and converted to digital audio files. The timing-only versions, rendered in Wood Blocks timbre (selected from the Musical Instrument Digital Interface, or MIDI, Instruments list), preserved the tempo, meter, and rhythmic structure of the original melodies without reference to pitch. A click track—in a different timbre (Bass Drum, MIDI)—provided a regular accompanying beat. The pitch-only versions, rendered in a synthetic flute timbre (Blown Bottle, MIDI) in the original key, preserved the original intervals between successive tones. All songs in this condition were presented in a similar pitch register. As a result, the pitch level of one song (from *Dora the Explorer*) was one octave lower than its melodic version. Moreover, all tones for each excerpt were of equal duration, and the tempo was normalized (to 90 beats per minute) across excerpts. Because the long-duration notes were shortened to match all other note durations, this manipulation disrupted the original tempo, rhythm, and meter. Excerpts in the original, instrumental, melodic, and timing-only conditions were approximately 15 s in duration. Because of the substitution of short-duration notes for long-duration notes in the pitch-only condition, those excerpts were approximately 10 s in duration. Musical notation for melodic and pitch-only versions of two of the theme songs are depicted in Figure 1. Melodic, timing-only, and pitch-only versions of these songs are available in Supplementary Materials.

### Procedure

Participants were tested individually and recruited on the basis of familiarity with at least two of the eight songs in the stimulus set. Prior to the test session, the experimenter asked the child and parent to choose the two TV shows that were most familiar to the child from the eight that were available. Before the first trial, pictorial representations of the two shows appeared simultaneously on the computer monitor, and each child responded accurately when the experimenter pointed to each picture in turn and asked, “Who's that? Tell me.” Children were told that they were going to hear songs from the two TV shows and that they were required to indicate “which show the song belongs to” by touching one of the pictures on the screen. The stimuli were presented in five blocks, corresponding to the five conditions. The conditions were presented in fixed order: original versions followed by instrumental, melodic, timing-only, and pitch-only versions. Each condition was preceded by two practice trials, one with each stimulus from that condition, to familiarize children with the materials and task. Children received automated feedback, as in the test trials. Before each practice trial, children heard pre-recorded instructions (“Listen to the music. Who's that? Show me.”) spoken by a woman in a child-directed manner.

Children proceeded to the test trials immediately after the two practice trials. Before each test trial, they heard shortened instructions (“Who's that? Show me.”). Stimuli within each block of test trials (2 shows X 5 repetitions of each song) were presented randomly for a total of 10 trials per block. After listening to each stimulus, participants responded by selecting the image from the corresponding show. They were free to respond as soon as they recognized the music. Correct responses were followed by the brief appearance of a smiley face on the monitor; incorrect responses resulted in a comparably brief blank screen.

## Results

**Figure 2:**
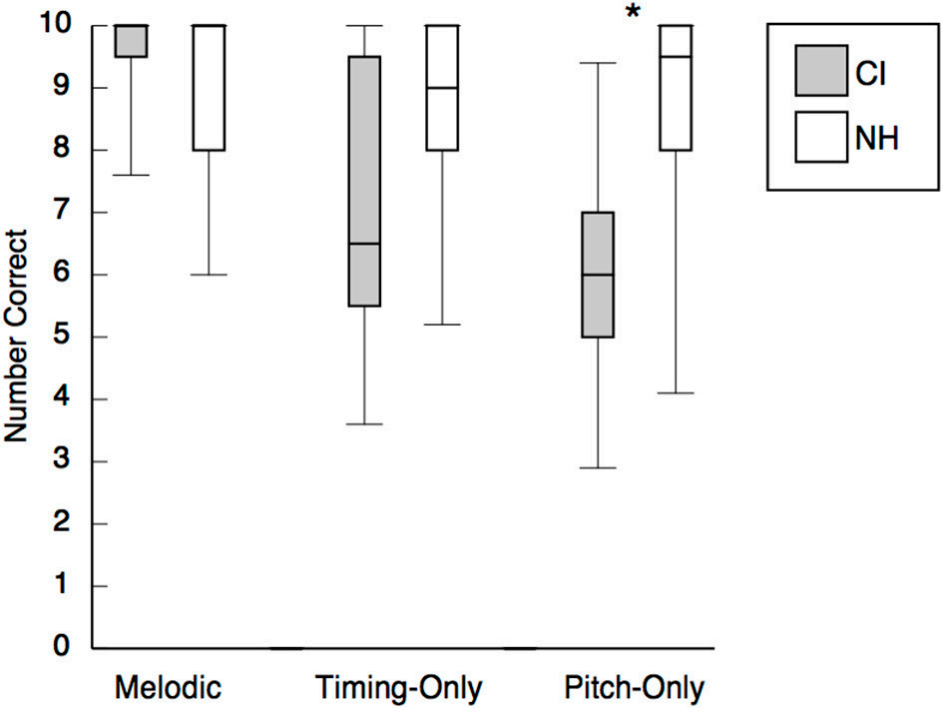
**Performance of child CI users and NH children in the melodic, timing-only, and pitch-only conditions**. NH children significantly outperformed child CI users in the pitch-only condition (indicated by the asterisk). Performance did not differ across conditions for the NH group, but CI users' performance in the melodic condition exceeded their performance in the timing-only and pitch-only conditions.

Preliminary analysis revealed high levels of performance for both groups of children (>91% correct) in the original and instrumental conditions, which established that all children recognized the two target songs with and without the lyrics, and confirmed that it was reasonable to proceed to our more specific questions about melody recognition. Performance of child CI users and NH listeners on the three conditions of principal interest is depicted in Figure 2. Because scores were distributed non-normally and sample sizes were small, nonparametric tests were used for all analyses. For the two groups of children (separately), we initially compared performance in each condition with chance levels (i.e., 5 correct on 10 trials with 2 response options per trial). If either group was performing at chance levels, approximately half of the children should have scores lower than chance (0–4), whereas the other half should be above chance (6–10). One-tailed sign tests (i.e., performance significantly below chance was uninterpretable) revealed that for the NH group, scores were above chance in all three conditions, *p*s ≤ 0.007. For the CI group, performance was above chance in the melodic condition, *p*s ≤ 0.008. In the timing-only condition, performance marginally exceeded chance levels, *p* = 0.063. In the pitch-only condition, child CI users performed at chance, *p* = 0.109.

The principal analyses examined performance differences among the melodic, timing-only, and pitch-only conditions. The use of nonparametric tests precluded formal examination of a two-way interaction between group and condition. For the NH group, a Friedman Analysis of Variance (ANOVA) for repeated measures revealed that performance was similar across the three conditions, *p* = 0.166. For the CI group, however, a second Friedman ANOVA confirmed that performance varied across conditions, *p* = 0.020. The CI group performed better in the melodic condition than in either the timing-only, *p* = 0.041, or the pitch-only, *p* = 0.018, condition, which did not differ, *p* = 0.599 (Wilcoxon signed-rank tests). Additional between-group comparisons indicated that performance of NH children and child CI users did not differ in the melodic, *p* = 0.417, and timing-only, *p* = 0.214, conditions, but the NH group outperformed the CI group in the pitch-only condition, *p* = 0.038.

**Figure 3:**
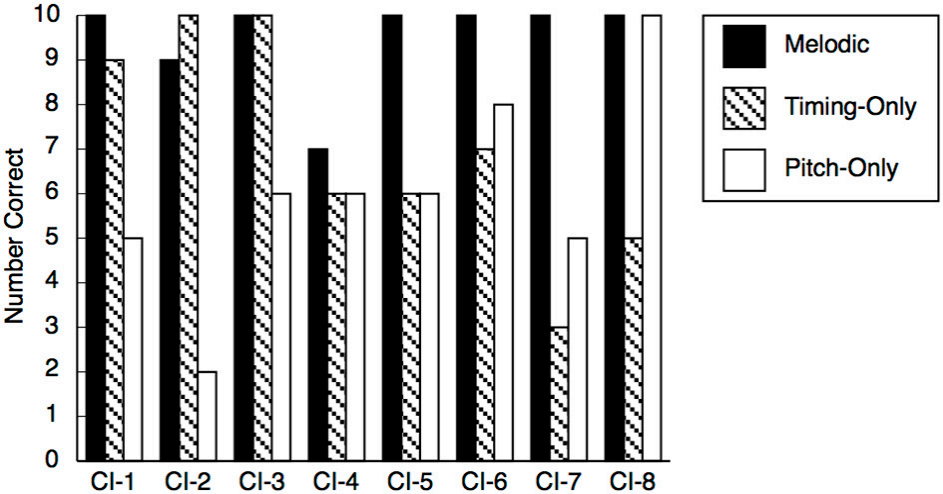
**Performance of individual CI users in the melodic, timing-only, and pitch-only conditions**.

Examination of individual CI user's performance (see Figure 3) revealed a more complex picture. Because the probability of guessing 8 or more answers out of 10 correctly is approximately 5% (binomial test, *p* = 0.055), only 3 CI participants (CI-4, CI-5, and CI-7) actually performed at chance levels in both timing-only and pitch-only conditions, with the youngest participant, CI-7, showing the poorest accuracy. Three child CI users achieved perfect (CI-2 and CI-3) or near-perfect (CI-1) scores in the timing-only condition, but those children were at chance in the pitch-only condition. In contrast, participant CI-8 was error-free in the pitch-only condition but performed very poorly in the timing-only condition. Participant CI-6 performed reasonably, albeit modestly (80%), in the pitch-only condition, achieving 70% accuracy in the timing-only condition. With the worst performer (CI-7) excluded, CI children's scores in these two conditions were negatively correlated, *r* = −0.733, *N* = 7, *p* = 0.030 (one-tailed), leading to speculation about a possible trade-off in the use of timing and pitch cues by young CI users. For the NH group, there was no such trade-off, *p* = 0.597.

Despite discrepancies in performance, individual data confirmed that the pitch-only condition generally presented greater problems for CI users than did the timing-only condition. In contrast, the majority of NH children performed comparably well in both conditions. We did not systematically document the strategies used by CI participants, but participant CI-1 commented that the difference in tempo was helpful in the timing-only condition (“This guy was faster than that guy”). Participant CI-3 reported linking the rhythm in the timing-only condition to the lyrics (“counted where the words were supposed to be”), which parallels young children's approach to song production (Welch et al., 1998).

## Discussion

The goal of the present investigation was to ascertain whether 5- to 7-year-old children with CIs and normally hearing (NH) children with comparable years of auditory experience could identify familiar TV songs from pitch or timing cues alone or in combination. First, it was necessary to confirm that young CI users could identify the original recordings as well as versions that preserved the instrumentation without the lyrics. In fact, CI listeners' performance in these conditions exceeded performance in earlier studies with TV theme songs (Mitani et al., 2007; Vongpaisal et al., 2009) and were comparable to the performance of NH children. Undoubtedly, the reduced cognitive demands of the present task, which featured two alternative responses rather than the three or four in previous studies and the inclusion of feedback, contributed to the exceptionally high performance levels and to the reduction of differences between NH children and child CI users.

Our principal focus was on three conditions: melodic, which provided pitch and timing cues, timing-only, which provided timing cues but no pitch cues, and pitch-only, which provided pitch cues but no timing cues. NH children performed equally well on all three conditions (approximately 85% correct) and almost as well as on the original recordings. The finding that young NH children can identify familiar songs from timing or pitch cues alone is unique to the present study. NH adults can do so, but they show substantial performance decrements on open-set song identification from pitch-only cues (49% correct) or timing-only cues (9% correct) as opposed to combined pitch and timing cues (approximately 90% correct; Hébert and Peretz, 1997). The present findings also contrast with performance decrements observed in a closed-set task (four-alternatives) when older NH children identified pop melodies from instrumental (original minus vocal) versions (85% correct) and melodic versions with combined pitch and timing cues (76% correct) as compared with the original versions (99% correct; Vongpaisal et al., 2006). As noted, the present tasks were considerably easier because of fewer response alternatives and the provision of feedback. Although pitch and timing cues may be integrated in young children's representations of familiar music (Overy et al., 2004), NH children in the present study had little difficulty using pitch or timing cues when presented alone.

In absolute terms, CI children performed better than NH children on the melodic versions of the excerpts (see Figure 2). In previous research, Japanese children of similar age were unable to recognize TV songs from comparable melodic cues (Mitani et al., 2007), and older Canadian children could do so but they performed more poorly on the melodic (approximately 37% correct) than on the original versions (65% correct; Vongpaisal et al., 2009). In both of the earlier studies, child CI users exhibited substantial performance decrements when the cues available at test differed from those available at home while watching TV. The authors claimed that child CI users' representations of music were less abstract than those of NH children, who were less affected by the elimination of timbre and texture cues. With the minimal cognitive demands of the current two-alternative task with feedback, children's performance was unaffected by such changes.

The marginally significant ability of young CI users to identify familiar music on the basis of timing cues is impressive when considered in conjunction with the small sample size, the absence of significant differences between CI and NH groups in this condition, and the significant group differences in the pitch-only condition. In fact, the bass drum click track, with its low frequency components, may have placed child CI users at a disadvantage because of the properties of their prostheses. The ability to make use of timing cues in music is consistent with studies of rhythm perception in adult CI users (Gfeller et al., 1997; Kong et al., 2004; Cooper et al., 2008). It is also consistent with child CI users' reliance on timing cues to differentiate one talker from another (Vongpaisal et al., 2010). In principle, child CI users and NH children could have used tempo to identify the timing-only patterns, but the extent to which they did so remains unclear. The fact that CI users' performance was well below ceiling on this condition argues against their use of tempo cues. Obviously, both hearing and deaf children would have much greater difficulty identifying the timing-only versions from three or more alternatives, and they are likely to be entirely unsuccessful on open-set tasks. Adults are similarly unsuccessful under these conditions, reporting that the pitch-only but not the timing-only versions sound familiar (Hébert and Peretz, 1997).

Child CI users' performance differed significantly from that of NH children only on the pitch-only versions, where their performance was at chance levels. In previous research, children with normal hearing outperformed child CI users even on canonical versions of familiar songs that included the original vocal and instrumental cues (Vongpaisal et al., 2006, 2009), which implies that the low difficulty and consequent high performance levels in the present study obscured ability differences between the groups. Task order was also confounded with task difficulty, which means that poorer performance on the later tasks could have resulted from fatigue or boredom. The overt behavior of all children, however, including CI users, indicated otherwise. Children were highly enthusiastic about the characters from their favorite TV programs, with some expressing disappointment when the test session ended.

Although child CI users' failure to identify songs by pitch cues alone implies that they relied primarily on timing cues, they performed significantly better on the melody versions, which had pitch and timing cues, than on the timing-only versions, which had timing cues alone. In other words, child CI users derived some benefit from pitch cues. Children whose program selections included *Dora the Explorer* could have used pitch register cues instead of or in addition to pitch contour cues in the melodic condition but not in the pitch-only condition. Because of the nature of their implant processor, child CI users, unlike NH children, may actually rely on some correlate of pitch such as timbre or loudness in performing these tasks.

In principle, children may not have recognized the songs initially in the melodic, timing-only, or pitch-only conditions, simply learning the correct responses from the feedback or reinforcement. This interpretation is highly unlikely for the NH children, who performed at or near ceiling on all conditions. For child CI users, modal performance on the melodic condition was 100% correct. We do not know of any evidence that children in this age range are capable of associating “unfamiliar” melodies with familiar pictures after one or two trials. With young children, reinforcement is commonly used to maintain children's interest and effort over the course of a test session.

Although the overall performance of child CI users was at chance levels on the pitch-only versions, one child (CI-8) achieved errorless performance on this and other versions except for the timing-only version, on which she performed poorly. Coding of fundamental frequency in current prostheses results in weak cues that impede normal music perception (Laneau et al., 2008). Perhaps the effective salience of pitch cues or their correlates could be enhanced by training, which would have implications well beyond the perception of music. There is suggestive evidence that music training improves the pitch-ranking ability of child CI users although performance remains uncorrelated with the actual distance between pitches (Chen et al., 2010). Again, the implication is that CI users use cues other than pitch in such pitch-ranking judgments.

The tendency of child CI users who performed well on the timing-only versions to perform poorly on the pitch-only versions raises the possibility of less flexibility in listening strategies relative to NH children, who readily switched from one strategy to another depending on the task. For listeners with CIs, listening in general is more effortful or cognitively demanding than it is for NH listeners (Pals et al., 2013), with music listening being particularly demanding. One consequence may be inefficient and ineffectual listening strategies.

In short, our findings suggest that prelingually deaf child CI users require fewer cues for the identification of familiar songs than one would expect from previous research with same-age and older children (Stordahl, 2002; Olszewski et al., 2005; Mitani et al., 2007; Vongpaisal et al., 2009; but see Hsiao, 2008). Child CI users in the present study were implanted earlier than children in previous studies and they had more advanced CI processors, so it is possible that these factors contributed to their success. We have no definitive evidence about child CI users' long-term representations of melodies, but such representations appear to include information about timing and perhaps coarse information related to pitch contour and pitch register. Further research with a larger sample is necessary to establish the contribution of experiential factors to child CI users' memory for melodies and for music in general. Recent evidence indicates that access to low-frequency hearing (i.e., relevant to musical pitch processing) in the pre-implant period results in better music perception and memory in the post-implant period (Hopyan et al., 2012). In light of the reported associations between music and well-being (Hanser, 2010) and between musical and non-musical skills (Schellenberg, 2004; Wong et al., 2007; Kraus and Chandrasekaran, 2010; Degé et al., 2011; Moreno et al., 2011), it is important to ascertain the extent to which music perception in child CI users can be improved by intervention or training.

## Author note

This research was approved by institutional ethics committees and funded by the Natural Sciences and Engineering Research Council of Canada. We are grateful to the participating children and their families, to Deanna Feltracco for assistance in data collection, and to Vicky Papaianno and Jerome Valero for their assistance and encouragement.

### Conflict of interest statement

The authors declare that the research was conducted in the absence of any commercial or financial relationships that could be construed as a potential conflict of interest.
